# Short-Term Impact of Digital Mental Health Interventions on Psychological Well-Being and Blood Sugar Control in Type 2 Diabetes Patients in Riyadh

**DOI:** 10.3390/healthcare12222257

**Published:** 2024-11-13

**Authors:** Abdulaziz M. Alodhialah, Ashwaq A. Almutairi, Mohammed Almutairi

**Affiliations:** 1 Department of Medical Surgical Nursing, College of Nursing, King Saud University, Riyadh 11421, Saudi Arabia; 2 School of Nursing & Midwifery, Monash University, Melbourne, VIC 3004, Australia; ashwaq.almutairi@monash.edu

**Keywords:** type 2 diabetes, digital mental health, glycemic control, psychological well-being, integrated care

## Abstract

Background: Type 2 diabetes (T2D) management is complicated by psychological factors, yet mental health interventions are not routinely integrated into diabetes care. This study investigated the impact of a digital mental health intervention on psychological well-being and glycemic control in T2D patients. Methods: A quasi-experimental study was conducted with 120 T2D patients divided into intervention (*n* = 60) and control (*n* = 60) groups. The intervention group received a one-month digital mental health intervention alongside standard care. Psychological well-being (PHQ-9, GAD-7, and DDS) and glycemic control (HbA1c) were assessed at baseline and post-intervention. Results: The intervention group showed significant improvements in HbA1c levels (−0.5%, *p* = 0.032), PHQ-9 (−3.1, *p* = 0.001), GAD-7 (−2.8, *p* = 0.006), and DDS (−7.7, *p* = 0.012) scores compared to the control group. Strong correlations were observed between psychological improvements and HbA1c reductions. Higher engagement with the digital platform was associated with greater improvements in both psychological and glycemic outcomes. Conclusions: Integrating digital mental health interventions into T2D care can significantly improve both psychological well-being and glycemic control. These findings support a more holistic approach to diabetes management that addresses both mental and physical health aspects.

## 1. Introduction

Diabetes, particularly type 2 diabetes (T2D), is one of the most prevalent chronic diseases globally, affecting millions of individuals and posing significant public health challenges [1]. Managing diabetes requires a holistic approach that extends beyond physical health, as psychological factors play a crucial role in influencing disease outcomes [2]. Numerous studies have established a strong correlation between diabetes and mental health conditions such as depression, anxiety, and diabetes-related distress [3]. These mental health challenges often arise due to the long-term nature of diabetes management, lifestyle modifications, and the emotional burden of living with a chronic illness [4,5]. However, despite growing evidence of the psychological impact of diabetes, mental health interventions are not routinely integrated into diabetes care plans [6,7].

This gap in diabetes care becomes particularly concerning as research shows that psychological distress in individuals with T2D can lead to poor treatment adherence, suboptimal glycemic control, and an overall reduction in quality of life [8,9]. Depression and anxiety, for instance, are associated with difficulties in maintaining recommended blood glucose levels, making it harder for individuals to manage their condition effectively [10]. In turn, uncontrolled diabetes exacerbates psychological distress, creating a cyclical relationship that adversely affects both mental and physical health outcomes [11]. Hence, the integration of mental health support into diabetes care is not only desirable but essential for improving patient outcomes [12]. However, traditional mental health interventions, such as in-person therapy or psychiatric support, are often inaccessible to many individuals due to the cost, location, and availability of mental health professionals [13].

In response to this accessibility issue, digital mental health interventions have emerged as a promising solution. The rapid advancement of technology has paved the way for mobile applications, teletherapy, online counseling, and cognitive behavioral therapy (CBT) platforms that can be delivered remotely and on-demand [14]. These interventions provide a more accessible, flexible, and scalable approach to mental healthcare, particularly for individuals managing chronic conditions like diabetes. Mobile health (mHealth) applications and platforms designed to improve mental well-being can be used alongside diabetes management tools, enabling patients to monitor their blood sugar levels while also receiving mental health support [15]. This integration of digital health technologies is particularly pertinent in the context of diabetes, as many digital interventions can target behaviors and psychological challenges directly related to diabetes management, such as stress, motivation, and self-efficacy [16].

Despite the growing interest in digital health interventions, there remains a significant gap in research regarding the effectiveness of these interventions in diabetes care, particularly in addressing both psychological and glycemic outcomes [17]. While studies have shown that digital interventions can be effective for treating depression and anxiety in the general population, their specific impact on individuals with T2D has not been thoroughly explored [18]. Moreover, most existing research focuses on either glycemic control or mental health outcomes separately, failing to investigate the potential benefits of integrating both components into a comprehensive care model [19]. This lack of integrated studies underscores the need for a more nuanced understanding of how digital mental health interventions can influence the dual outcomes of psychological well-being and glycemic control [20].

One of the key issues in the current literature is the heterogeneity of interventions studied. Digital mental health interventions encompass a wide range of tools, including mobile applications, online CBT, telepsychiatry, and even virtual reality therapy [21]. Each of these tools may have varying levels of effectiveness depending on the individual’s specific needs, the severity of their mental health symptoms, and the level of diabetes-related distress they experience [22]. Furthermore, there is a lack of consensus on which mental health interventions are most appropriate for different subgroups within the diabetic population. For instance, individuals newly diagnosed with diabetes may experience different psychological challenges than those who have been living with the disease for many years, and tailored interventions may be needed [23]. Therefore, the field lacks standardized protocols or clear guidelines on the best practices for integrating digital mental health interventions into diabetes care.

Another critical gap is the understanding of long-term effectiveness and adherence to digital interventions. Although many digital tools show promise in short-term studies, the sustainability of their impact remains uncertain [24]. In diabetes care, where long-term adherence to lifestyle changes and medical regimens is crucial, understanding how patients engage with digital mental health tools over time is essential [25]. Additionally, there is a gap in understanding how healthcare providers perceive and incorporate digital mental health interventions into diabetes care [26]. While patients may have access to a range of digital tools, their effectiveness often depends on the involvement of healthcare professionals who can provide guidance, support, and monitoring [27]. Yet, little research has been conducted on how healthcare teams integrate these tools into routine diabetes care, the potential barriers they face, and how training and resources can be improved to facilitate this process. This is particularly relevant in the context of managing T2D, where ongoing, multidisciplinary care is crucial for success. The role of nurses, dietitians, endocrinologists, and mental health professionals in supporting the use of digital tools must be better understood to create an integrated care model [28].

### 1.1. Aim of the Study

The aim of this study is to evaluate the effectiveness of integrating digital mental health interventions into diabetes care, focusing on their impact on both psychological well-being and glycemic control in individuals with type 2 diabetes. The study seeks to explore how digital tools, such as mobile apps and online therapy, can support mental health while improving diabetes management outcomes, including treatment adherence and blood glucose regulation.

### 1.2. Research Questions

1. What is the impact of integrating digital mental health interventions on psychological well-being (e.g., depression, anxiety, and diabetes-related distress) in individuals with type 2 diabetes?
2. How does the use of digital mental health interventions influence glycemic control and treatment adherence in patients with type 2 diabetes?
3. What factors affect the long-term adherence and engagement with digital mental health tools in individuals with type 2 diabetes?
4. How do healthcare providers perceive and integrate digital mental health interventions into routine diabetes care?

## 2. Materials and Methods

### 2.1. Study Design

This study employed a quasi-experimental pretest–post-test design with a one-month follow-up period. The participants were divided into the following two groups: an intervention group that received a digital mental health intervention alongside standard diabetes care and a control group that continued with standard diabetes care alone. The study was designed to assess the impact of the digital mental health intervention on both psychological well-being and glycemic control over the course of one month. Data collection took place at the following two points: baseline (before the intervention) and one month after the intervention.

### 2.2. Study Setting

The study was conducted in Riyadh, Saudi Arabia, at multiple healthcare facilities known for their expertise in diabetes care, including outpatient clinics and primary care centers. These facilities were selected to ensure diversity in the study population, representing individuals from various socioeconomic backgrounds and healthcare access levels. Riyadh was chosen due to its status as a major metropolitan area, allowing the recruitment of a sufficient number of participants and access to the necessary healthcare resources.

### 2.3. Sample and Sampling Procedure

The study targeted adult patients diagnosed with type 2 diabetes (T2D) who were receiving care at healthcare facilities in Riyadh, Saudi Arabia. A purposive sampling method was used to recruit a total of 120 participants. This method allowed for the selection of individuals who met specific inclusion criteria, ensuring that the sample was representative of the population relevant to the study’s objectives. The study aimed to include participants who were likely to benefit from the integration of digital mental health interventions with their diabetes management.

### 2.4. Inclusion Criteria

Participants were required to meet the following inclusion criteria.

#### 2.4.1. Diagnosis of Type 2 Diabetes

Participants needed to have been diagnosed with T2D for at least one year to ensure familiarity with diabetes management and to assess the potential impact of psychological distress on long-term diabetes care.

#### 2.4.2. Mild-To-Moderate Psychological Symptoms

Individuals presenting with mild-to-moderate symptoms of depression, anxiety, or diabetes-related distress, as determined by their baseline psychological assessments using the PHQ-9, GAD-7, and DDS scales, were included. This criterion ensured that the sample consisted of individuals who might benefit from mental health interventions.

#### 2.4.3. Age 18 and Above

Participants needed to be adults (18 years or older) to ensure that they could provide informed consent and were legally able to participate in the study.

#### 2.4.4. Access to Digital Devices

Participants needed to have regular access to smartphones or computers with Internet connectivity. This criterion was necessary to ensure that participants in the intervention group could engage with the digital mental health platform used in the study.

### 2.5. Exclusion Criteria

Participants were excluded if they met any of the following criteria.

#### 2.5.1. Severe Psychiatric Disorders

Individuals with severe psychiatric conditions, such as schizophrenia, bipolar disorder, or any condition that required intensive psychiatric care, were excluded to ensure that the study focused on those with mild-to-moderate psychological symptoms. This also minimized the risk of exacerbating serious conditions without providing adequate mental healthcare.

#### 2.5.2. Inability to Use Digital Tools

Individuals who were unable to use digital devices independently, either due to a lack of familiarity or physical limitations that prevented regular use of a smartphone or computer, were excluded. This criterion ensured that participants in the intervention group could fully engage with the digital mental health platform.

#### 2.5.3. Pregnancy

Pregnant women were excluded from the study due to potential confounding effects of pregnancy on glycemic control and mental health, which might have influenced the study’s results.

#### 2.5.4. Significant Comorbidities

Participants with significant comorbidities, such as advanced cardiovascular disease, chronic kidney disease, or other serious health conditions, were excluded. These conditions could independently impact both psychological well-being and glycemic control, potentially confounding the study’s findings.

### 2.6. Sampling Procedure

The recruitment process took place at selected healthcare facilities in Riyadh that specialized in diabetes care. Healthcare providers at these facilities assisted in identifying potential participants by reviewing medical records to find individuals who met the inclusion criteria. Eligible patients were then approached by the research team, who explained the study objectives and procedures in detail.

Once potential participants expressed interest in the study, they underwent an initial screening process. During this screening, the research team administered the PHQ-9, GAD-7, and DDS assessments to determine their psychological status. Patients meeting the criteria for mild-to-moderate symptoms were invited to participate. Participants were given detailed information about the study’s purpose, procedures, and potential risks and benefits, and written informed consent was obtained from all participants.

### 2.7. Sample Size Determination

A sample size of 120 participants (60 in the intervention group and 60 in the control group) was determined based on a power analysis conducted prior to the study. The analysis aimed to detect a significant difference in psychological well-being and glycemic control between the intervention and control groups with an 80% power and a 5% significance level (*p* < 0.05). Given the anticipated effect sizes from previous studies on similar interventions, this sample size was sufficient to account for potential dropout rates and ensure the statistical reliability of the study results.

### 2.8. Group Allocation

Participants were non-randomly assigned to either the intervention group (*n* = 60) or the control group (*n* = 60) based on their willingness to participate in the digital intervention and their access to digital devices. Although random assignment was not possible due to practical constraints, efforts were made to match participants across the two groups based on demographic factors such as age, gender, and baseline HbA1c levels. This matching process helped ensure that the groups were comparable and minimized the potential for confounding variables to influence the results.

### 2.9. Participant Traits

The final sample included individuals from diverse demographic backgrounds, reflecting the population of Riyadh. Participants varied in age, socioeconomic status, education level, and diabetes management experience. Both male and female participants were included, providing a broad representation of adults living with type 2 diabetes in the region. This diversity in the sample was intended to enhance the generalizability of the study’s findings, making them applicable to a wide range of individuals with type 2 diabetes in Saudi Arabia and beyond.

### 2.10. Data Collection Tools

In this study, several standardized and validated tools were used to collect data on psychological well-being, glycemic control, and treatment adherence. These tools were chosen for their reliability, validity, and widespread use in clinical and research settings, particularly among individuals with type 2 diabetes. Detailed descriptions of each tool are provided below:

1. The Patient Health Questionnaire-9 (PHQ-9) was developed by Drs. Robert L. Spitzer, Janet B.W. Williams, and Kurt Kroenke in collaboration with Pfizer Inc. as part of the larger PRIME-MD diagnostic tool [29]. The PHQ-9 is a self-administered tool designed to screen for the presence and severity of depression in clinical settings. It consists of nine items, each corresponding to the nine diagnostic criteria for major depressive disorder outlined in the Diagnostic and Statistical Manual of Mental Disorders, Fourth Edition (DSM-IV). Each item is rated on a 4-point Likert scale, ranging from 0 (not at all) to 3 (nearly every day), with total scores ranging from 0 to 27. Higher scores indicate greater severity of depression. The scoring system categorizes depression severity as follows: 0–4 represents minimal depression, 5–9 indicates mild depression, 10–14 reflects moderate depression, 15–19 suggests moderately severe depression, and 20–27 indicates severe depression. An example of a question from the PHQ-9 is: “Over the last two weeks, how often have you been bothered by little interest or pleasure in doing things?” The responses range from “not at all” to “nearly every day.” The PHQ-9 has demonstrated excellent reliability and validity, with a Cronbach’s alpha reliability score typically reported between 0.86 and 0.89, indicating high internal consistency. The tool is widely used in both clinical practice and research due to its brevity, ease of use, and strong psychometric properties.
2. The Generalized Anxiety Disorder-7 (GAD-7) was developed by Dr. Robert L. Spitzer and his colleagues as a brief screening tool to assess the severity of generalized anxiety disorder (GAD) symptoms [30]. This self-report questionnaire is widely used in both clinical and research settings due to its ease of administration and strong psychometric properties. The GAD-7 consists of seven items, each reflecting core symptoms of anxiety as defined by the DSM-IV criteria, such as feeling nervous, inability to control worry, and trouble relaxing. Each item is rated on a 4-point Likert scale, with responses ranging from 0 (“not at all”) to 3 (“nearly every day”). The total score ranges from 0 to 21, with higher scores indicating greater severity of anxiety symptoms. Scores are categorized as follows: 0–4 (minimal anxiety), 5–9 (mild anxiety), 10–14 (moderate anxiety), and 15–21 (severe anxiety). An example of an item from the GAD-7 is: “Feeling nervous, anxious, or on edge”. This prompt requires respondents to indicate how often they have experienced this feeling in the past two weeks. The tool’s internal consistency is high, with a Cronbach’s alpha reliability score of 0.92, demonstrating strong reliability.
3. The Diabetes Distress Scale (DDS) was developed by Polonsky and colleagues in 2005 to assess the unique emotional burdens and concerns specific to living with diabetes [31]. It is a 17-item self-report questionnaire that measures the distress associated with managing diabetes across the following four dimensions: emotional burden, physician-related distress, regimen-related distress, and interpersonal distress. Each item is rated on a 6-point Likert scale, ranging from 1 (no distress) to 6 (serious distress), with higher scores indicating greater distress. The scoring system involves calculating the average score across all items, with a score of 2.0 or lower indicating little to no distress, between 2.0 and 2.9 representing moderate distress, and 3.0 or higher suggesting significant distress that may require attention. An example of the content includes questions like, “Feeling overwhelmed by the demands of living with diabetes”, which falls under emotional burden. The DDS has been shown to have strong internal consistency, with Cronbach’s alpha reliability scores ranging from 0.87 to 0.93 across its subscales, making it a reliable tool for assessing diabetes-related emotional distress.

### 2.11. Intervention (Data Collection Procedure)

#### Intervention Design

The intervention group received a comprehensive official and widely used digital mental health platform that is free and available globally is MindShift CBT, developed by Anxiety Canada. The platform was accessible via smartphones and computers, ensuring flexibility and convenience for participants. It integrated several core features aimed at reducing psychological distress, improving coping mechanisms, and enhancing self-management behaviors.

The intervention was delivered over a one-month period, during which participants were encouraged to engage with the platform at least twice a week for a minimum of 30 min per session. The platform’s content was designed to be user-friendly, allowing participants to engage with the modules at their own pace and according to their personal schedules.

### 2.12. Components of the the Intervention

#### Cognitive Behavioral Therapy (CBT) Modules

These modules provided structured, evidence-based therapeutic exercises aimed at addressing negative thought patterns, managing diabetes-related stress, and fostering positive emotional responses. The CBT content focused on key areas such as:

- Identifying and challenging automatic negative thoughts.
- Learning to reframe unhelpful thinking patterns.
- Coping with stress associated with diabetes management, such as medication adherence and blood glucose monitoring.
- Enhancing self-efficacy in managing the emotional burden of living with a chronic illness.

The CBT exercises included text-based prompts, interactive activities, and self-assessment quizzes. Participants were asked to reflect on their emotions and thoughts related to diabetes management and implement the CBT techniques in their daily lives.

### 2.13. Mindfulness and Relaxation Exercises

Guided mindfulness sessions were available to help participants manage stress and anxiety. These exercises included:

- Breathing exercises to promote relaxation and reduce physiological responses to stress.
- Guided imagery and progressive muscle relaxation to relieve tension and calm the mind.
- Mindfulness meditation exercises, which encouraged participants to focus on the present moment and practice non-judgmental awareness of their thoughts and feelings.

The mindfulness exercises were designed to be short and easily integrated into the daily routines of the participants. The goal was to help participants build resilience against the stressors associated with chronic disease management and improve their emotional regulation.

### 2.14. Educational Resources on Diabetes Self-Management

This educational content provided via email or whats app booklet aimed to improve participants’ understanding of diabetes and encourage better self-management. Topics included:

- Blood glucose monitoring techniques.
- The importance of medication adherence.
- Dietary guidelines and physical activity recommendations.
- Strategies for coping with diabetes-related emotional distress and social support networks.

These educational resources were delivered in the form of short articles, videos, and quizzes to engage participants and enhance their knowledge of diabetes self-management. The educational content was tailored to address both the physical and emotional challenges of diabetes, promoting a more holistic approach to disease management.

### 2.15. Control Group Procedure

Participants in the control group did not have access to the digital mental health platform. They continued with their usual diabetes care regimen as prescribed by their healthcare providers, which included regular consultations, medication adherence, and self-monitoring of blood glucose. No additional mental health support or interventions were provided to this group during the study period.

### 2.16. Data Collection Procedure

Data were collected at the following two key time points: baseline (before the intervention) and one-month post-intervention. The following steps outline the detailed process for data collection:

#### 2.16.1. Baseline Data Collection (Pre-Intervention)

At the start of the study, all participants—both in the intervention and control groups—underwent baseline assessments to establish their initial psychological and glycemic status. Data were collected using the following standardized tools:

#### 2.16.2. Psychological Well-Being

Self-administered questionnaires, the PHQ-9 for depression, GAD-7 for anxiety, and the Diabetes Distress Scale (DDS) for diabetes-related emotional distress, were filled out by the participants during their initial visit or via a secure online form.

#### 2.16.3. Glycemic Control

Hemoglobin A1c (HbA1c) tests were conducted at the baseline visit to assess long-term blood glucose levels. Additionally, participants were instructed to begin daily self-monitoring of blood glucose (SMBG) using a digital glucometer. They were provided with a logbook or an app to record their fasting blood glucose levels.

#### 2.16.4. Treatment Adherence

The Morisky Medication Adherence Scale (MMAS-8) was used to assess participants’ adherence to their diabetes medication regimens. This scale was also self-administered at the baseline assessment.

### 2.17. One-Month Follow-Up Data Collection (Post-Intervention)

After one month of engaging with the intervention, participants returned for a follow-up assessment. The same tools were used to collect post-intervention data, allowing for direct comparison with baseline measurements. These included:

- Psychological Well-being: Participants once again completed the PHQ-9, GAD-7, and DDS questionnaires to assess any changes in their mental health status after the intervention.
- Glycemic Control: HbA1c tests were repeated to evaluate any changes in long-term blood glucose control. Participants also submitted their daily SMBG logs for review.
- Treatment Adherence: The MMAS-8 was administered again to assess any changes in medication adherence over the one-month period.

### 2.18. Monitoring of Engagement with the Digital Platform

In addition to the scheduled assessments, data on participants’ engagement with the digital mental health platform were collected throughout the intervention period. This included the frequency of logins, the completion of CBT modules and mindfulness exercises, and the time spent on each activity. These engagement metrics were analyzed to explore their relationship with the psychological and glycemic outcomes.

### 2.19. Control Group Follow-Up

Participants in the control group also returned for the one-month follow-up assessment. They completed the same psychological well-being questionnaires, underwent HbA1c testing, and provided their SMBG logs. The MMAS-8 was administered again to assess any changes in their medication adherence.

### 2.20. Summary of Data Collection Points

- Time Point 1 (Baseline): All participants completed the PHQ-9, GAD-7, DDS, and MMAS-8, and underwent HbA1c testing. SMBG logging then began.
- Time Point 2 (One-Month Post-Intervention): All participants repeated the psychological assessments, HbA1c testing, and MMAS-8. SMBG logs were collected for review.

### 2.21. Statistical Analysis

All data were analyzed using SPSS version 25, with both descriptive and inferential statistics applied to assess the intervention’s impact on psychological well-being and glycemic control. Descriptive statistics, including means, standard deviations, and frequency distributions, were calculated for variables such as age, gender, baseline HbA1c levels, and psychological well-being measures (PHQ-9, GAD-7, and DDS), as well as treatment adherence (MMAS-8). These summaries helped establish baseline characteristics and ensure comparability between the intervention and control groups. Prior to conducting inferential tests, the normality of the data was assessed using the Shapiro–Wilk test, as well as visual inspections of histograms and Q-Q plots. For within-group comparisons of pre- and post-intervention data, paired t-tests were used to evaluate changes in psychological well-being (PHQ-9, GAD-7, and DDS), HbA1c levels, and medication adherence (MMAS-8). Independent t-tests were used to compare the mean post-intervention scores between the intervention and control groups to assess the differential effects of the digital mental health intervention. Additionally, a linear regression analysis was conducted to explore the relationship between participants’ engagement with the digital mental health platform and their improvements in psychological well-being and glycemic control outcomes. A significance level of *p* < 0.05 was set for all statistical tests to determine the significance of the findings.

### 2.22. Ethical Considerations

The study was conducted in strict accordance with the ethical principles outlined in the Declaration of Helsinki to ensure the rights, dignity, and well-being of all participants. Ethical approval was obtained from the Institutional Review Board (IRB) of King Saud University in Riyadh, Saudi Arabia (IRB approval number: 24-684 on 1 August 2024), and written informed consent was obtained from all participants prior to their inclusion in the study. Participants were informed of their right to withdraw from the study at any time without repercussions, and they were assured that their participation or withdrawal would not affect their standard of care. Confidentiality and privacy were maintained by anonymizing all of the data and securely storing them to prevent unauthorized access. All data were encrypted, and only the research team had access to identifiable information. In the event that any participant exhibited worsening psychological symptoms during the study, they were referred to appropriate mental health services for further evaluation and support. Additionally, the digital intervention adhered to privacy regulations, ensuring that all interactions on the platform were secure and confidential.

## 3. Results

### Participant Characteristics

Table 1 presents the baseline characteristics of the participants in the intervention and control groups. Both groups were well-matched, with no significant differences in age (mean 54.3 years in the intervention group vs. 55.1 years in the control group, *p* = 0.573) or gender distribution (53.3% males in the intervention group vs. 50% in the control group, *p* = 0.723). Similarly, the baseline HbA1c levels were comparable (8.4% in the intervention group vs. 8.3% in the control group, *p* = 0.745), along with the initial psychological well-being scores (PHQ-9, GAD-7, and DDS) and treatment adherence (MMAS-8). The similarity between groups at baseline supports the validity of the comparisons made after the intervention.

Table 2 demonstrates the significant improvements in the intervention group compared to the control group after one month. The intervention group showed a substantial reduction in HbA1c levels (from 8.4% to 7.9%, *p* = 0.032), whereas the control group remained largely unchanged (from 8.3% to 8.2%, *p* = NS). Psychological well-being also improved markedly in the intervention group, with PHQ-9 scores decreasing from 11.5 to 8.4 (*p* = 0.001), GAD-7 scores dropping from 10.3 to 7.5 (*p* = 0.006), and DDS scores reducing from 34.2 to 26.5 (*p* = 0.012). In contrast, the control group experienced minimal improvements in these scores, highlighting the positive effect of the digital mental health intervention on both mental health and glycemic outcomes.

Table 3 shows the correlations between the changes in HbA1c and psychological well-being in the intervention group. Significant positive correlations were observed between the reductions in HbA1c and improvements in PHQ-9 (r = 0.452, *p* < 0.05), GAD-7 (r = 0.385, *p* < 0.05), and DDS (r = 0.411, *p* < 0.05). This suggests that better glycemic control was associated with improvements in mental health. Additionally, strong correlations between the psychological well-being measures themselves (PHQ-9, GAD-7, and DDS) indicate that participants experiencing improvements in one domain of mental health often showed corresponding improvements in other domains

Table 4 presents the logistic regression analysis results, showing that changes in PHQ-9 (OR = 1.289, *p* = 0.011), GAD-7 (OR = 1.217, *p* = 0.027), and DDS (OR = 1.245, *p* = 0.018) were significant predictors of glycemic improvement (HbA1c reduction by at least 0.5%). Baseline HbA1c was also a significant predictor (OR = 1.366, *p* = 0.045), indicating that those with higher baseline HbA1c levels were more likely to achieve glycemic improvement. Age and gender were not significant predictors, suggesting that the mental health improvements were the primary factors driving better glycemic outcomes in the intervention group.

Table 5 highlights the effect of the engagement frequency with the digital intervention on outcomes. Participants with high engagement (using the platform at least twice a week) showed significantly greater reductions in HbA1c (−0.8 vs. −0.4, *p* = 0.001), PHQ-9 (−4.2 vs. −2.6, *p* = 0.003), GAD-7 (−3.5 vs. −1.8, *p* = 0.008), and DDS scores (−7.9 vs. −4.1, *p* = 0.004) compared to those with low engagement. These findings underscore the importance of regular interaction with the digital mental health platform to maximize its benefits, both in terms of psychological well-being and glycemic control.

Table 6 reports the results of the post-intervention satisfaction survey among participants in the intervention group. A significant majority of participants (87%) reported being either “very satisfied” (47%) or “satisfied” (40%) with the digital mental health platform, indicating a high level of acceptance and satisfaction with the intervention. Only a small percentage (3%) reported dissatisfaction, and no participants indicated being “very dissatisfied”. This high level of satisfaction suggests that the digital intervention was well-received, which likely contributed to the positive outcomes observed in the intervention group.

## 4. Discussion

The findings of this study provide compelling evidence for the efficacy of integrating digital mental health interventions into the care of patients with type 2 diabetes (T2D). The results demonstrate significant improvements in both psychological well-being and glycemic control among participants who engaged with the digital mental health platform compared to those who received standard care alone. This integrated approach addresses the complex interplay between mental health and diabetes management, offering a promising avenue for enhancing overall patient outcomes.

### 4.1. Glycemic Control Improvements

One of the most striking outcomes of this study was the substantial reduction in HbA1c levels observed in the intervention group. The decrease from 8.4% to 7.9% (*p* = 0.032) over just one month is clinically significant and suggests that addressing psychological factors through digital interventions can have a meaningful impact on glycemic control. This finding aligns with previous research indicating that psychological interventions can improve glycemic control in diabetes patients [32,33,34]. The magnitude of this improvement is particularly noteworthy given the short duration of the intervention, suggesting that digital mental health tools may offer a rapid and effective means of enhancing diabetes management [35].

The mechanism behind this improvement may be related to enhanced self-management behaviors, reduced diabetes-related distress, and improved medication adherence, all of which are known to influence glycemic control [36,37]. By providing accessible psychological support and educational resources, the digital platform may have empowered participants to better engage with their diabetes care regimen [38]. This highlights the potential of digital interventions to bridge the gap between mental health support and diabetes self-management, addressing a critical need in comprehensive diabetes care [39].

### 4.2. Psychological Well-Being Enhancements

The significant improvements in psychological well-being measures (PHQ-9, GAD-7, and DDS) in the intervention group further underscore the effectiveness of the digital mental health platform. These results are consistent with meta-analyses showing that digital mental health interventions can effectively reduce symptoms of depression and anxiety in various populations, including those with chronic illnesses [40]. The reduction in diabetes-related distress (DDS) is particularly noteworthy, as high levels of diabetes distress have been associated with poor glycemic control and reduced self-care behaviors [41,42].

The observed improvements across multiple psychological domains suggest that the digital intervention provided comprehensive mental health support tailored to the specific needs of T2D patients [43]. This multifaceted approach may be more effective than interventions targeting single aspects of mental health, as it addresses the complex psychological challenges often faced by individuals managing chronic conditions like diabetes [44].

In addition to the observed improvements in psychological well-being and glycemic control, it is worth considering the potential physiological mechanisms that may explain this relationship [45]. One possibility is that mental health interventions, such as the digital CBT platform used in this study, may reduce the activation of the sympathetic nervous system. Chronic activation of the sympathetic nervous system is known to trigger gluconeogenesis and lipolysis, which, in turn, increase blood glucose levels [46]. By reducing stress and anxiety, the mental health intervention could potentially downregulate these pathways, thereby leading to better glycemic control. This hypothesis aligns with research showing that stress reduction can positively impact metabolic health, particularly in individuals managing chronic conditions like diabetes [47].

### 4.3. Interplay Between Mental Health and Diabetes Management

The strong correlations observed between improvements in psychological well-being and reductions in HbA1c levels provide insight into the interplay between mental health and diabetes management. This relationship has been noted in previous studies, which have shown that addressing psychological factors can lead to improvements in diabetes outcomes [48]. Our findings extend this understanding by demonstrating that these benefits can be achieved through digital interventions, potentially offering a more accessible and scalable approach to integrated diabetes care.

The bidirectional nature of this relationship is particularly interesting. While improved mental health may lead to better diabetes self-management, the converse may also be true: better glycemic control could contribute to reduced psychological distress. This reciprocal relationship underscores the importance of addressing both mental and physical health aspects simultaneously in diabetes care [49].

### 4.4. Predictors of Glycemic Improvement

The logistic regression analysis revealed that changes in psychological well-being measures were significant predictors of glycemic improvement. This suggests that the mental health component of the intervention played a crucial role in improving diabetes outcomes, rather than the improvement being solely due to increased attention to diabetes management. This finding supports the growing body of evidence advocating for the integration of mental healthcare into diabetes management [50].

The significance of baseline HbA1c as a predictor of improvement indicates that individuals with poorer initial glycemic control may stand to benefit the most from such interventions. This has important implications for targeting digital mental health interventions to those who may derive the greatest benefit, potentially optimizing resource allocation in healthcare settings [51].

### 4.5. Engagement and Outcomes

The impact of engagement frequency on outcomes highlights the importance of regular interaction with digital health interventions. Participants who engaged with the platform more frequently experienced greater improvements in both psychological and glycemic outcomes. This dose–response relationship has been observed in other digital health interventions and underscores the need for strategies to promote sustained engagement with these tools [52].

The observed relationship between engagement and outcomes raises important questions about how to optimize digital interventions for maximum benefit. Future research should explore methods to enhance user engagement and identify the optimal frequency of interaction [53]. This may involve personalized reminders, gamification elements, or adaptive content that evolves based on user interaction patterns. Additionally, investigating the factors that contribute to high engagement could inform the design of more effective digital health interventions [54].

### 4.6. Patient Satisfaction and Acceptance

The high level of satisfaction reported by participants in the intervention group is encouraging and suggests that digital mental health interventions can be well-received by patients with T2D. This acceptance is crucial for the successful implementation of such interventions in real-world clinical settings. The positive reception may be attributed to the convenience, privacy, and personalization offered by digital platforms, which have been identified as key factors in patient engagement with digital health tools [55,56].

The high satisfaction rates also suggest that digital interventions may help overcome some of the barriers associated with traditional mental health support, such as stigma or limited access to mental health professionals. This could potentially lead to the increased uptake of mental health support among T2D patients, addressing an often unmet need in this population [57].

While this study demonstrates the effectiveness of integrating digital mental health interventions in improving both psychological well-being and glycemic control, it does not fully explore the underlying mechanisms responsible for these improvements. One potential mechanism is that stress reduction, facilitated by the intervention, may reduce sympathetic nervous system activation, thereby decreasing gluconeogenesis and improving blood sugar levels. Further research is required to investigate these physiological pathways and their role in the observed improvements.

### 4.7. Limitations and Future Directions

This study has several limitations that should be acknowledged. First, the sample size (*n* = 120) may limit the generalizability of the findings. While the sample size was sufficient for the initial power analysis, larger studies are necessary to confirm these preliminary results and further validate the observed improvements in psychological well-being and glycemic control.

Second, the duration of the study was relatively short, lasting only one month. Although significant improvements were observed in this period, type 2 diabetes is a chronic condition, and longer follow-up periods are required to assess the sustainability of the intervention’s effects. Future research should extend the duration of follow-up to determine whether the psychological and glycemic improvements can be maintained over time.

Third, participants were not randomly assigned to the intervention and control groups, which introduces the potential for selection bias. Although efforts were made to match participants based on demographic factors such as age, gender, and baseline HbA1c levels, a randomized controlled trial (RCT) design would have provided stronger evidence of the intervention’s effectiveness by minimizing bias.

Additionally, the study focused on patients with mild-to-moderate psychological symptoms, which limits the generalizability of the findings to individuals with more severe mental health conditions. The results may not apply to patients with serious psychiatric disorders, and future studies should include participants with a broader range of psychological conditions to explore the efficacy of digital mental health interventions in more diverse patient populations.

The study was conducted in Riyadh, Saudi Arabia, and although efforts were made to recruit participants from diverse backgrounds, the findings may not be fully generalizable to other populations or healthcare settings. Cultural, socioeconomic, and healthcare access differences could influence the outcomes in different regions. Future research should replicate the study in different countries and healthcare systems to better assess the broader applicability of the findings.

Finally, while the study demonstrated significant improvements in psychological well-being and glycemic control, the underlying mechanisms responsible for these improvements were not fully explored. It is possible that the mental health intervention reduced stress and anxiety, leading to lower activation of the sympathetic nervous system and subsequent improvements in glycemic control. However, further research is needed to investigate these physiological pathways in more details.

### 4.8. Implications for Clinical Practice and Health Policy

The findings of this study have several important implications for clinical practice and health policy. First, they suggest that healthcare providers should consider incorporating digital mental health tools into their care plans for patients with T2D, particularly those showing signs of psychological distress. This could involve recommending specific digital platforms as part of a comprehensive treatment plan or integrating digital mental health screening and support into existing diabetes management programs.

Second, the results highlight the need for a more holistic approach to diabetes management that addresses both physical and mental health aspects of the condition. This integrated approach could potentially lead to better overall outcomes and improved quality of life for patients with T2D [41]. Healthcare systems may need to adapt to support this integrated model, potentially requiring changes in provider training, care coordination, and reimbursement structures.

From a health policy perspective, the study provides evidence to support the allocation of resources towards the development and implementation of digital mental health interventions in diabetes care. The potential for these interventions to improve both psychological and glycemic outcomes could lead to reduced healthcare costs and improved population health outcomes in the long term [42]. Policymakers should consider incentivizing the adoption of evidence-based digital mental health tools in diabetes care settings and supporting further research in this area.

### 4.9. Future Research Directions

While this study provides valuable insights, it also opens up several avenues for future research. Larger-scale randomized controlled trials are needed to further validate these findings and explore the long-term effects of digital mental health interventions in diabetes care. These studies should include diverse patient populations and healthcare settings to enhance the generalizability.

Investigating the cost-effectiveness of digital mental health interventions in diabetes care would provide valuable information for healthcare systems considering their implementation. This could involve comparing the costs and outcomes of digital interventions with traditional mental health support or standard diabetes care alone.

Research into the mechanisms underlying the observed improvements in glycemic control and psychological well-being would deepen our understanding of how digital interventions affect diabetes management. This could involve more detailed monitoring of self-management behaviors, medication adherence, and physiological stress markers.

Exploring ways to personalize digital mental health interventions for individual patients with T2D could enhance their effectiveness. This might involve using machine learning algorithms to tailor content and recommendations based on user characteristics and engagement patterns.

Finally, investigating the potential of integrating digital mental health interventions with other digital health tools, such as continuous glucose monitors or insulin pumps, could lead to more comprehensive and effective diabetes management systems.

## 5. Conclusions

This study provides important preliminary evidence that integrating digital mental health interventions into diabetes care can positively impact both psychological well-being and glycemic control in patients with type 2 diabetes. While the one-month duration of this study demonstrates early improvements, longer-term studies are necessary to fully elucidate the underlying mechanisms, such as the potential effects on metabolic pathways and sustained behavioral changes. Future research should extend the duration of follow-up to determine whether these improvements can be maintained over time and to explore the physiological and psychological processes that contribute to the observed outcomes. As the use of digital tools becomes more prevalent, understanding their long-term benefits will be essential for developing comprehensive and sustainable diabetes care strategies.

## Figures and Tables

**Table 1:** Baseline characteristics of the study participants.

Variable	Intervention Group (*n* = 60)	Control Group (*n* = 60)	*p*-Value
Age (years, mean ± SD)	54.3 ± 8.2	55.1 ± 7.9	0.573
Gender (Male, *n* %)	32 (53.3%)	30 (50%)	0.723
HbA1c (% mean ± SD)	8.4 ± 1.3	8.3 ± 1.2	0.745
PHQ-9 (mean ± SD)	11.5 ± 3.2	11.7 ± 3.1	0.682
GAD-7 (mean ± SD)	10.3 ± 2.7	10.1 ± 2.8	0.798
DDS (mean ± SD)	34.2 ± 8.1	33.9 ± 7.9	0.854
MMAS-8 (mean ± SD)	6.2 ± 1.0	6.3 ± 1.1	0.651

**Table 2:** Pre- and post-intervention changes in psychological well-being and glycemic control.

Variable	Intervention Group (Pre-)	Intervention Group (Post-)	Control Group (Pre-)	Control Group (Post-)	*p*-Value (Between Groups)
HbA1c (% mean ± SD)	8.4 ± 1.3	7.9 ± 1.2	8.3 ± 1.2	8.2 ± 1.3	0.032
PHQ-9 (mean ± SD)	11.5 ± 3.2	8.4 ± 2.8	11.7 ± 3.1	11.1 ± 3.2	0.001
GAD-7 (mean ± SD)	10.3 ± 2.7	7.5 ± 2.4	10.1 ± 2.8	9.7 ± 2.9	0.006
DDS (mean ± SD)	34.2 ± 8.1	26.5 ± 7.3	33.9 ± 7.9	32.9 ± 7.7	0.012
MMAS-8 (mean ± SD)	6.2 ± 1.0	7.1 ± 0.9	6.3 ± 1.1	6.4 ± 1.1	0.043

**Table 3:** Correlation between HbA1c and psychological well-being measures in the intervention group.

Variable	HbA1c	PHQ-9	GAD-7	DDS
HbA1c	1	0.452 *	0.385 *	0.411 *
PHQ-9	0.452 *	1	0.634 **	0.529 **
GAD-7	0.385 *	0.634 **	1	0.501 *
DDS	0.411 *	0.529 **	0.501 *	1

* = significant relation; ** = highly significant.

**Table 4:** Logistic regression analysis predicting glycemic control improvement (reduction in HbA1c by ≥0.5%).

Variable	B	SE	OR	95% CI for OR	*p*-Value
PHQ-9	0.254	0.094	1.289	1.072–1.542	0.011
GAD-7	0.196	0.086	1.217	1.031–1.436	0.027
DDS	0.219	0.089	1.245	1.048–1.478	0.018
Age	−0.032	0.021	0.969	0.930–1.009	0.128
Gender (Male = 1, Female = 0)	0.487	0.416	1.628	0.715–3.707	0.246
Baseline HbA1c	0.312	0.156	1.366	1.008–1.850	0.045

**Table 5:** Impact of engagement frequency with the digital intervention on outcomes.

Engagement Level	HbA1c (Mean ± SD)	PHQ-9 (Mean ± SD)	GAD-7 (Mean ± SD)	DDS (Mean ± SD)
High Engagement (*n* = 32)	−0.8 ± 0.3	−4.2 ± 1.5	−3.5 ± 1.4	−7.9 ± 3.1
Low Engagement (*n* = 28)	−0.4 ± 0.2	−2.6 ± 1.1	−1.8 ± 1.2	−4.1 ± 2.7
*p*-Value	0.001	0.003	0.008	0.004

**Table 6:** Post-intervention satisfaction survey results (intervention group).

Satisfaction Level	*n* (%)
Very satisfied	28 (47%)
Satisfied	24 (40%)
Neutral	6 (10%)
Dissatisfied	2 (3%)
Very dissatisfied	0 (0%)

## Data Availability

All data are available within the manuscript.

## References

[1] Khan M.A.B., Hashim M.J., King J.K., Govender R.D., Mustafa H., Al Kaabi J. (2019). Epidemiology of Type 2 Diabetes—Global Burden of Disease and Forecasted Trends. J. Epidemiol. Glob. Health.

[2] Kalra S., Jena B., Yeravdekar R. (2018). Emotional and Psychological Needs of People with Diabetes. Indian J. Endocrinol. Metab..

[3] Sugandh F., Chandio M., Raveena F., Kumar L., Karishma F., Khuwaja S., Memon U.A., Bai K., Kashif M., Varrassi G. (2023). Advances in the Management of Diabetes Mellitus: A Focus on Personalized Medicine. Cureus.

[4] Ahmad F., Joshi S.H. (2023). Self-Care Practices and Their Role in the Control of Diabetes: A Narrative Review. Cureus.

[5] Vadakkiniath I.J. (2023). Prevalence and Correlates of Stress, Anxiety, and Depression in Patients with Chronic Diseases: A Cross-Sectional Study. Middle East Curr. Psychiatry.

[6] Young-Hyman D., de Groot M., Hill-Briggs F., Gonzalez J.S., Hood K., Peyrot M. (2016). Psychosocial Care for People with Diabetes: A Position Statement of the American Diabetes Association. Diabetes Care.

[7] Snoek F.J. (2022). Mental Health in Diabetes Care. Time to Step Up. Front. Clin. Diabetes Healthc..

[8] Adu M.D., Malabu U.H., Malau-Aduli A.E.O., Malau-Aduli B.S. (2019). Enablers and Barriers to Effective Diabetes Self-Management: A Multi-National Investigation. PLoS ONE.

[9] Davies M.J., Aroda V.R., Collins B.S., Gabbay R.A., Green J., Maruthur N.M., Rosas S.E., Del Prato S., Mathieu C., Mingrone G. (2022). Management of Hyperglycemia in Type 2 Diabetes, 2022. A Consensus Report by the American Diabetes Association (ADA) and the European Association for the Study of Diabetes (EASD). Diabetes Care.

[10] Basiri R., Seidu B., Rudich M. (2023). Exploring the Interrelationships between Diabetes, Nutrition, Anxiety, and Depression: Implications for Treatment and Prevention Strategies. Nutrients.

[11] Burns R.J., Deschênes S.S., Schmitz N. (2015). Cyclical Relationship between Depressive Symptoms and Diabetes Distress in People with Type 2 Diabetes Mellitus: Results from the Montreal Evaluation of Diabetes Treatment Cohort Study. Diabet. Med..

[12] Garrett C., Doherty A. (2014). Diabetes and Mental Health. Clin. Med..

[13] Alavi N., Moghimi E., Stephenson C., Gutierrez G., Jagayat J., Kumar A., Shao Y., Miller S., Yee C.S., Stefatos A. (2023). Comparison of Online and In-Person Cognitive Behavioral Therapy in Individuals Diagnosed with Major Depressive Disorder: A Non-Randomized Controlled Trial. Front. Psychiatry.

[14] Lattie E.G., Stiles-Shields C., Graham A.K. (2022). An Overview of and Recommendations for More Accessible Digital Mental Health Services. Nat. Rev. Psychol..

[15] Eberle C., Löhnert M., Stichling S. (2021). Effectiveness of Disease-Specific MHealth Apps in Patients with Diabetes Mellitus: Scoping Review. JMIR mHealth uHealth.

[16] Shaban M.M., Sharaa H.M., Amer F.G.M., Shaban M. (2024). Effect of Digital Based Nursing Intervention on Knowledge of Self-Care Behaviors and Self-Efficacy of Adult Clients with Diabetes. BMC Nurs..

[17] Morris T., Aspinal F., Ledger J., Li K., Gomes M. (2023). The Impact of Digital Health Interventions for the Management of Type 2 Diabetes on Health and Social Care Utilisation and Costs: A Systematic Review. PharmacoEconomic Open.

[18] Varela-Moreno E., Carreira Soler M., Guzmán-Parra J., Jódar-Sánchez F., Mayoral-Cleries F., Anarte-Ortíz M.T. (2022). Effectiveness of EHealth-Based Psychological Interventions for Depression Treatment in Patients with Type 1 or Type 2 Diabetes Mellitus: A Systematic Review. Front. Psychol..

[19] AlHaqwi A.I., Amin M.M., AlTulaihi B.A., Abolfotouh M.A. (2023). Impact of Patient-Centered and Self-Care Education on Diabetes Control in a Family Practice Setting in Saudi Arabia. Int. J. Environ. Res. Public Health.

[20] Taher R., Hsu C.-W., Hampshire C., Fialho C., Heaysman C., Stahl D., Shergill S., Yiend J. (2023). The Safety of Digital Mental Health Interventions: Systematic Review and Recommendations. JMIR Ment. Health.

[21] Philippe T.J., Sikder N., Jackson A., Koblanski M.E., Liow E., Pilarinos A., Vasarhelyi K. (2022). Digital Health Interventions for Delivery of Mental Health Care: Systematic and Comprehensive Meta-Review. JMIR Ment. Health.

[22] Wojujutari A.K., Idemudia E.S., Ugwu L.E. (2024). Psychological Resilience Mediates the Relationship between Diabetes Distress and Depression among Persons with Diabetes in a Multi-Group Analysis. Sci. Rep..

[23] De Wit M., Gajewska K.A., Goethals E.R., McDarby V., Zhao X., Hapunda G., Delamater A.M., DiMeglio L.A. (2022). ISPAD Clinical Practice Consensus Guidelines 2022: Psychological Care of Children, Adolescents and Young Adults with Diabetes. Pediatr. Diabetes.

[24] Ward J., Davies G., Dugdale S., Elison S., Bijral P. (2017). Achieving Digital Health Sustainability: Breaking Free and CGL. Int. J. Health Gov..

[25] Sands D.Z. (2023). Beyond the EHR: How Digital Health Tools Foster Participatory Health and Self-Care for Patients with Diabetes. Am. J. Med. Open.

[26] Racey M., Whitmore C., Alliston P., Cafazzo J.A., Crawford A., Castle D., Dragonetti R., Fitzpatrick-Lewis D., Jovkovic M., Melamed O.C. (2023). Technology-Supported Integrated Care Innovations to Support Diabetes and Mental Health Care: Scoping Review. JMIR Diabetes.

[27] Yeung A.W.K., Torkamani A., Butte A.J., Glicksberg B.S., Schuller B., Rodriguez B., Ting D.S.W., Bates D., Schaden E., Peng H. (2023). The Promise of Digital Healthcare Technologies. Front. Public Health.

[28] Gómez-Velasco D.V., Almeda-Valdes P., Martagón A.J., Galán-Ramírez G.A., Aguilar Salinas C.A. (2019). Empowerment of Patients with Type 2 Diabetes: Current Perspectives. Diabetes, Metab. Syndr. Obes. Targets Ther..

[29] Kroenke K., Spitzer R.L., Williams J.B.W. (2001). The PHQ-9. J. Gen. Intern. Med..

[30] Spitzer R.L., Kroenke K., Williams J.B.W., Löwe B. (2006). A Brief Measure for Assessing Generalized Anxiety Disorder. Arch. Intern. Med..

[31] Polonsky W.H., Fisher L., Earles J., Dudl R.J., Lees J., Mullan J., Jackson R.A. (2005). Assessing Psychosocial Distress in Diabetes. Diabetes Care.

[32] Plakas S., Mastrogiannis D., Mantzorou M., Adamakidou T., Fouka G., Bouziou A., Tsiou C., Morisky D.E. (2016). Validation of the 8-Item Morisky Medication Adherence Scale in Chronically Ill Ambulatory Patients in Rural Greece. Open J. Nurs..

[33] Winkley K., Upsher R., Stahl D., Pollard D., Brennan A., Heller S.R., Ismail K. (2020). Psychological Interventions to Improve Glycemic Control in Adults with Type 2 Diabetes: A Systematic Review and Meta-Analysis. BMJ Open Diabetes Res. Care.

[34] Kebede M.M., Zeeb H., Peters M., Heise T.L., Pischke C.R. (2018). Effectiveness of Digital Interventions for Improving Glycemic Control in Persons with Poorly Controlled Type 2 Diabetes: A Systematic Review, Meta-Analysis, and Meta-Regression Analysis. Diabetes Technol. Ther..

[35] Ni Y., Ma L., Li J. (2021). Effects of Mindfulness-based Intervention on Glycemic Control and Psychological Outcomes in People with Diabetes: A Systematic Review and Meta-analysis. J. Diabetes Investig..

[36] Hessler D., Fisher L., Glasgow R.E., Strycker L.A., Dickinson L.M., Arean P.A., Masharani U. (2014). Reductions in Regimen Distress Are Associated with Improved Management and Glycemic Control Over Time. Diabetes Care.

[37] Fayed A., AlRadini F., Alzuhairi R.M., Aljuhani A.E., Alrashid H.R., Alwazae M.M., Alghamdi N.R. (2022). Relation between Diabetes Related Distress and Glycemic Control: The Mediating Effect of Adherence to Treatment. Prim. Care Diabetes.

[38] ElSayed N.A., Aleppo G., Aroda V.R., Bannuru R.R., Brown F.M., Bruemmer D., Collins B.S., Hilliard M.E., Isaacs D., Johnson E.L. (2023). 1. Improving Care and Promoting Health in Populations: Standards of Care in Diabetes—2023. Diabetes Care.

[39] Ernawati U., Wihastuti T.A., Utami Y.W. (2021). Effectiveness of Diabetes Self-Management Education (Dsme) in Type 2 Diabetes Mellitus (T2Dm) Patients: Systematic Literature Review. J. Public Health Res..

[40] Andrews B., Klein B., Van Nguyen H., Corboy D., McLaren S., Watson S. (2023). Efficacy of a Digital Mental Health Biopsychosocial Transdiagnostic Intervention With or Without Therapist Assistance for Adults with Anxiety and Depression: Adaptive Randomized Controlled Trial. J. Med. Internet Res..

[41] Alyahyawi N.Y., Alrifay R.M., Albadi N.A., Alqahtani M.Y., Alzahrani R.M., Nazer B.A., Alghamdi J.S., Bahattab J.A. (2021). The Impact of Diabetes Distress on the Glycemic Control Among Adolescents and Youth with Type 1 Diabetes in Two Tertiary Centers, Jeddah, Saudi Arabia. Cureus.

[42] Ibrahim A., Rida A., Dakroub D., Cherri S., Fahs H., Hammoud J., Hallit S., El Khatib S., Altyar A.E., Abdel-Daim M.M. (2023). Association between Diabetes Distress and Sociodemographic and/or Socioeconomic Factors among Adults: A Cross-Sectional Study. Heliyon.

[43] Massey C.N., Feig E.H., Duque-Serrano L., Wexler D., Moskowitz J.T., Huffman J.C. (2019). Well-Being Interventions for Individuals with Diabetes: A Systematic Review. Diabetes Res. Clin. Pract..

[44] Carswell C., Coventry P.A., Brown J.V.E., Alderson S.L., Double K., Gilbody S., Holt R.I.G., Jacobs R., Lister J., Osborn D. (2023). Development of a Supported Self-Management Intervention for People with Severe Mental Illness and Type 2 Diabetes: Theory and Evidence-Based Co-Design Approach. J. Med. Internet Res..

[45] Yang X., Li Z., Sun J. (2020). Effects of Cognitive Behavioral Therapy–Based Intervention on Improving Glycaemic, Psychological, and Physiological Outcomes in Adult Patients with Diabetes Mellitus: A Meta-Analysis of Randomized Controlled Trials. Front. Psychiatry.

[46] Jenkinson E., Knoop I., Hudson J.L., Moss-Morris R., Hackett R.A. (2022). The Effectiveness of Cognitive Behavioural Therapy and Third-wave Cognitive Behavioural Interventions on Diabetes-related Distress: A Systematic Review and Meta-analysis. Diabet. Med..

[47] Clemente-Suárez V.J., Martín-Rodríguez A., Redondo-Flórez L., López-Mora C., Yáñez-Sepúlveda R., Tornero-Aguilera J.F. (2023). New Insights and Potential Therapeutic Interventions in Metabolic Diseases. Int. J. Mol. Sci..

[48] Pérez-Fernández A., Fernández-Berrocal P., Gutiérrez-Cobo M.J. (2023). The Relationship between Well-being and HbA1c in Adults with Type 1 Diabetes: A Systematic Review. J. Diabetes.

[49] Alzoubi A., Abunaser R., Khassawneh A., Alfaqih M., Khasawneh A., Abdo N. (2018). The Bidirectional Relationship between Diabetes and Depression: A Literature Review. Korean J. Fam. Med..

[50] Thangiah G., Johar H., Ismail R., Reininghaus U., Bärnighausen T., Thurairajasingam S., Reidpath D., Su T.T. (2022). Diabetes Treatment and Mental Illness: A Call for an Integrated Health Care System in Underserved Semi-Rural Malaysia. Int. J. Environ. Res. Public Health.

[51] Sherwani S.I., Khan H.A., Ekhzaimy A., Masood A., Sakharkar M.K. (2016). Significance of HbA1c Test in Diagnosis and Prognosis of Diabetic Patients. Biomark. Insights.

[52] Nelson L.A., Spieker A.J., Mayberry L.S., McNaughton C., Greevy R.A. (2021). Estimating the Impact of Engagement with Digital Health Interventions on Patient Outcomes in Randomized Trials. J. Am. Med. Inform. Assoc..

[53] Saleem M., Kühne L., De Santis K.K., Christianson L., Brand T., Busse H. (2021). Understanding Engagement Strategies in Digital Interventions for Mental Health Promotion: Scoping Review. JMIR Ment. Health.

[54] Kim H.K. (2024). Attraction and Achievement as 2 Attributes of Gamification in Healthcare: An Evolutionary Concept Analysis. J. Educ. Eval. Health Prof..

[55] Madanian S., Nakarada-Kordic I., Reay S., Chetty T. (2023). Patients’ Perspectives on Digital Health Tools. PEC Innov..

[56] Eppes E.V., Augustyn M., Gross S.M., Vernon P., Caulfield L.E., Paige D.M. (2023). Engagement With and Acceptability of Digital Media Platforms for Use in Improving Health Behaviors Among Vulnerable Families: Systematic Review. J. Med. Internet Res..

[57] Moghimi E., Knyahnytska Y., Omrani M., Nikjoo N., Stephenson C., Layzell G., Frederic Simpson A.I., Alavi N. (2022). Benefits of Digital Mental Health Care Interventions for Correctional Workers and Other Public Safety Personnel: A Narrative Review. Front. Psychiatry.

